# Metabolic Heterogeneity of Tumor Cells and its Impact on Colon Cancer Metastasis: Insights from Single-Cell and Bulk Transcriptome Analyses

**DOI:** 10.7150/jca.94630

**Published:** 2024-06-03

**Authors:** Yiwen Jia, Guangming Feng, Siyuan Chen, Wenhao Li, Zeguo Jia, Jian Wang, Hongxia Li, Shaocheng Hong, Fu Dai

**Affiliations:** 1 Department of Gastroenterology, The Third Affiliated Hospital of Anhui Medical University (Hefei first people's Hospital), Hefei, China.; 2 Department of Oncology, The Third Affiliated Hospital of Anhui Medical University (Hefei first people's Hospital), Hefei, 230032, China.; 3 Department of Pathology, The Third Affiliated Hospital of Anhui Medical University (Hefei first people's Hospital), Hefei, 230032, China.; 4 Department of Pulmonology, The First Affiliated Hospital of Anhui Medical University, Hefei, 230032, China.; 5 Department of Endocrinology, The First Affiliated Hospital of Anhui Medical University, Hefei, 230032, China.

**Keywords:** Tumor metabolic, Colorectal cancer, Single-cell, Tumor heterogeneity, Metastasis, Tumor microenvironment

## Abstract

**Background:** Metabolic reprogramming plays a crucial role in the development of colorectal cancer (CRC), influencing tumor heterogeneity, the tumor microenvironment, and metastasis. While the interaction between metabolism and CRC is critical for developing personalized treatments, gaps remain in understanding how tumor cell metabolism affects prognosis. Our study introduces novel insights by integrating single-cell and bulk transcriptome analyses to explore the metabolic landscape within CRC cells and its mechanisms influencing disease progression. This approach allows us to uncover metabolic heterogeneity and identify specific metabolic genes impacting metastasis, which have not been thoroughly examined in previous studies.

**Methods:** We sourced microarray and single-cell RNA sequencing datasets from the Gene Expression Omnibus (GEO) and bulk sequencing data for CRC from The Cancer Genome Atlas (TCGA). We employed Gene Set Variation Analysis (GSVA) to assess metabolic pathway activity, consensus clustering to identify CRC-specific transcriptome subtypes in bulkseq, and rigorous quality controls, including the exclusion of cells with high mitochondrial gene expression in scRNA seq. Advanced analyses such as AUCcell, infercnvCNV, Non-negative Matrix Factorization (NMF), and CytoTRACE were utilized to dissect the cellular landscape and evaluate pathway activities and tumor cell stemness. The hdWGCNA algorithm helped identify prognosis-related hub genes, integrating these findings using a random forest machine learning model.

**Results:** Kaplan-Meier survival curves identified 21 significant metabolic pathways linked to prognosis, with consensus clustering defining three CRC subtypes (C3, C2, C1) based on metabolic activity, which correlated with distinct clinical outcomes. The metabolic activity of the 13 cell subpopulations, particularly the epithelial cell subpopulation with active metabolic levels, was evaluated using AUCcell in scRNA seq. To further analyze tumor cells using infercnv, NMF disaggregated these cells into 10 cellular subpopulations. Among these, the C2 subpopulation exhibited higher stemness and tended to have a poorer prognosis compared to C6 and C0. Conversely, the C8, C3, and C1 subpopulations demonstrated a higher level of the five metabolic pathways, and the C3 and C8 subpopulations tended to have a more favorable prognosis. hdWGCNA identified 20 modules, from which we selected modules primarily expressed in high metabolic tumor subgroups and highly correlated with clinical information, including blue and cyan. By applying variable downscaling of RF to a total of 50 hub genes, seven gene signatures were obtained. Furthermore, molecules that were validated to be protective in GEO were screened alongside related molecules, resulting in the identification of prognostically relevant molecules such as UQCRFS1 and GRSF1. Additionally, the expression of GRSF1 was examined in colon cancer cell lines using qPCR and phenotypically verified by *in vitro* experiments.

**Conclusion:** Our findings emphasize that high activity in specific metabolic pathways, including pyruvate metabolism and the tricarboxylic acid cycle, correlates with improved colon cancer outcomes, presenting new avenues for metabolic-based therapies. The identification of hub genes like GRSF1 and UQCRFS1 and their link to favorable metabolic profiles offers novel insights into tumor neovascularization and metastasis, with significant clinical implications for targeting metabolic pathways in CRC therapy.

## Introduction

Colorectal cancer (CRC) is a global health concern, ranking as the fourth most commonly diagnosed and third deadliest cancer worldwide. To reduce the burden of CRC, targeted interventions are necessary, including primary prevention in low-income settings and early detection in high-income settings 1, 2. Molecular profiling has become increasingly important in therapeutic strategies for CRC patients, emphasizing the need for personalized and targeted approaches 3, 4. However, challenges persist in improving prognosis and reducing mortality, particularly in advanced stages of the disease 5, 6.

Metabolic disorders play a crucial role in cellular physiology and are intricately linked to the pathogenesis of various diseases, including CRC. Metabolic dysregulation enhances tumor cell metastasis and affects their ability to adapt to different microenvironments 7, 8. Therefore, understanding the metabolic mechanisms underlying CRC metastasis is vital for identifying potential targets and developing therapeutic strategies. CRC exhibits significant tumor heterogeneity, posing challenges in treatment. Different subpopulations of tumor cells may have distinct metabolic pathways, influencing their response to therapy and metastatic potential. The tumor microenvironment, consisting of immune cells, vascular endothelial cells, and fibroblasts, also plays a critical role in CRC development 9-11. The complex interactions between these cell types influence metabolic regulation and tumor progression. However, there are still gaps in our knowledge regarding cell-cell interactions and metabolic regulation in the CRC microenvironment.

Advancements in single-cell technology have provided new insights into tumor heterogeneity and the tumor microenvironment in CRC 12, 13. Through meticulous single-cell analysis, we can gain a deeper understanding of metabolic states, signaling cascades, and interaction patterns among different cell subpopulations. These efforts will undoubtedly unravel the mechanistic basis of metabolic abnormalities in CRC development, leading to breakthroughs in customized therapies 14, 15. In conclusion, exploring the interaction between metabolism and CRC is crucial for understanding tumor heterogeneity, the tumor microenvironment, and metastasis, as well as for developing personalized therapeutic strategies.

However, the impact of tumor cell metabolism on patient prognosis remains poorly understood. To address this question, our study combines single-cell and bulk transcriptome analyses to gain insights into the metabolic landscape within tumor cells and its potential mechanisms affecting colon cancer progression. We utilized microarray and single-cell RNA sequencing datasets from the Gene Expression Omnibus (GEO) and extensive sequencing data from The Cancer Genome Atlas (TCGA) database. The metabolic pathway activities were evaluated using Gene Set Variation Analysis (GSVA), and a consensus clustering approach was employed to identify CRC-specific bulk transcriptome isoforms. Our study identified 21 prognostically significant metabolic pathways, including the tricarboxylic acid cycle and pyruvate metabolism, and classified TCGA CRC samples into three subtypes based on their metabolic pathway activity. Additionally, we identified key genes associated with metabolism that affect colon cancer metastasis.

Our results revealed distinct metabolic activities among 13 cell subpopulations, with epithelial cells exhibiting the highest metabolic pathway activity. Furthermore, we identified different programs within tumor cells, assessed the level of cell stemness and CNV, and determined the pseudotime trajectory of CRC cells. Integrating extensive sequencing data and machine learning methods, we identified key genes associated with prognosis and validated the expression and function of GRSF1 in various CRC cell lines. These findings highlight the metabolic heterogeneity of tumor cells and the association between specific metabolic pathways and CRC prognosis, providing valuable insights for the development of personalized treatment strategies.

## Materials and Methods

### Data acquisition and pre-processing of CRC samples from public database

A total of 1631 colorectal cancer (CRC) samples were collected for expression profiling from the Gene Expression Omnibus (GEO) and The Cancer Genome Atlas (TCGA) databases. From the GEO database, five microarray datasets (GSE17536, GSE17537, GSE29621, GSE39582, and GSE72970) were downloaded using the GEOquery R software package 16-20. Gene expression data in transcripts per million (TPM) format from the TCGA-COAD and TCGA-READ cohorts, as well as somatic mutation data processed by MuTect2, were acquired from the UCSC Xena browser (https://xenabrowser.net/datapages/). For the colorectal cancer patient dataset sourced from GEO and TCGA, we conducted additional screening and preserved the data of patients with survival information for future analyses, encompassing 613 cases from TCGA and 1006 from GEO. Additionally, a single-cell RNA sequencing (scRNA-seq) dataset (GSE188711) consisting of six CRC samples (three from the left colon and three from the right colon) was obtained from the GEO database 21.

To process the data, the ComBat algorithm from the sva R package was used to merge the five GEO microarray datasets, creating a comprehensive dataset called meta-GEO 22. This step aimed to minimize potential batch effects arising from non-biotechnological biases among the different datasets. For the scRNA-seq dataset, the Seurat software package (version 4.3.0) was employed 23. Each sample was read using the Read10X function, and seurat objects were created with the parameters min.cells = 3 and min.features = 200. Further quality control measures were applied to the cells, including screening for genes detected in the cells (ranging from a minimum of 500 to a maximum of 5000), percentage of mitochondrial genes (ranging from 0% to 20%), and percentage of hemoglobin genes (ranging from 0% to 1%). In addition, we performed online analyses based on single-cell data obtained from the TISCH2 database 24.

### Integration of scRNA-seq, dimensionality reduction clustering and cellular annotation

The scRNA data was normalized using the "LogNormalize" method in the "NormalizeData" function. After normalization, the top 2000 highly variable genes were identified using the "FindVariableFeatures" function. To reduce the dimensionality of the scRNA-seq data, principal component analysis (PCA) was performed based on these 2000 highly variable genes. To address potential batch effects between samples, cell integration was performed using the R package harmony. Subsequently, cell clustering was performed using the "FindClusters" and "FindResolution" function with a resolution of 0.8. The clustering algorithm groups cells based on the similarity of their gene expression patterns to identify distinct cell populations in the scRNA-seq dataset. The clustering results were visualized using Unified Mobility Approximation and Projection (UMAP), a dimensionality reduction technique that projects high-dimensional data onto a two-dimensional plane. To identify cell types, we annotated cells based on previous literature or known marker genes.

### Consensus clustering and etimation of TME

Unsupervised cluster analysis was applied to identify different metabolic modification patterns based on 21 prognostically relevant metabolism-related pathway activities and to classify CRC patients for further analysis. This analysis was performed using the unsupervised clustering "Pam" method based on the Euclidean and Ward linkage by using the "ConsensuClusterPlus" R software package and 1000 replications to ensure stability of the classification 25. Transcriptional differences between three prognostically relevant metabolic CRC subtypes compared using a PCA approach. We used the MCPcounter algorithm of the Multi-omics Immuno-Oncology Biological Research (IOBR) package to compare the amounts of immune cell infiltration for CRC samples 26, 27. In addition, stromal scores and tumor purity were compared between different CRC molecular typologies using the ESTIMATE algorithm 28.

### Kaplan-Meier survival analysis

To assess differences in survival outcomes, the construction of Kaplan-Meier survival curves was used. For survival analysis, the survival software package (version 3.5.0) and the survminer package (version 0.4.9) were used, resulting in the identification of metabolic pathways or molecules associated with prognosis and the comparison of prognostic differences across CRC molecular subtypes.

### Geneset functional analysis for bulkseq

Enrichment of metabolic pathway gene sets in CRC patient samples in bulkseq can be assessed by GSVA 29. Subsequent differences in metabolic pathways were obtained using limma (version 3.54.0) package calculations, and thresholds of adj.P.Val <0.05 as well as logFC>0.1 were adopted. Fast Genome Enrichment Analysis (FGSEA) was performed according to the MsigDB download H.all.v7.2. using the fGSEA (version 1.24.0) R package 30. Enrichment analysis was performed for terms related to gene ontology biological process (GOBP) for DEGs between C3 and C1. In addition to this classical biological signaling pathway activity was scored for each sample using progeny (version 1.17.3) 31.

### Epithelial (tumor) cell state and the chromosomal copy‑number variations (CNV) estimation

First, the InferCNV (version 1.14.2) package was utilized to calculate copy number variations (CNV) in all epithelial cells 32. Neutrophils were chosen as a reference for this analysis. Using K-means clustering, epithelial cells displaying significant chromosomal copy number variations were identified as tumor cells. The CNV score was then calculated based on established methodologies from previous studies 33. Following the identification of tumor cells based on their chromosomal copy number variations, a non-negative matrix factorization (NMF) algorithm was employed to downscale all tumor cells 34. For scoring gene sets from single-cell sequencing data, we used the AUCell package, an algorithm that calculates gene set activity at single-cell resolution. Based on the set of metabolism-related genes obtained from the Kyoto Encyclopedia of Genes and Genomes (KEGG) database and the eight tumor cell states identified by Dalia Barkley et al., the AUCell was employed to assess the activity of metabolism-related signatures and tumor cell state in each malignant cell 35. In addition the CYTOTRACE algorithm was used to assess the stemness of cellular Clusters obtained by NMF 36.

### Pseudotime analysis of CRC maligent cells

Trajectory analysis was performed using Monocle (version 2.26.0) to understand the cellular changes that occur during differentiation of different CRC cells 37. Monocle objects were first constructed using the "newCellDataSet" function, then cells were ordered by filtering highly variable genes and down-dimensioned using the "DDRTree" algorithm to construct temporal trajectories. Subsequently, differentially expressed genes along the pseudotime were detected using the "differentialGeneTest" function and visualized by pseudotime heatmap. To functionally annotate these differential genes, we used Metascape (http://metascape.org). Metascape is an online tool that integrates various databases and algorithms for gene ontology and pathway enrichment analysis.

### Cell communication analysis in TME

To infer cell-cell interactions between tumor cells and immune/stromal cell types, we employed CellChat (version 1.6.1) software 38. This tool utilizes the expression of ligand-receptor pairs from the CellChatDB.human database, which contains information on "Secreted Signaling”. Using a default-based workflow, potential receptor-ligand pairs were identified and cellular communication networks with fewer than 10 cells were filtered out. Subsequently, receptor-ligand pairs associated with signaling pathways crucial for cellular interactions were extracted for visualization.

### HdWGCNA analysis

To construct a scale-free network at the single-cell level, high dimensional weighted gene co-expression network analysis (hdWGCNA) was utilized 39. This analysis was performed using the R package hdWGCNA (version 0.1.1.9010). The first step involved setting a threshold for the scale-free topology model fit. A threshold value greater than 0.8 was chosen to ensure a scale-free network structure. Next, a soft threshold of 14 was selected to achieve optimal connectivity within the network. This parameter influences the strength of correlations between genes and determines the modules or clusters of co-expressed genes.To score the TCGA COAD/READ cohort with the obtained modules, GSVA was employed. Correlations between the modules and phenotypic traits were evaluated using Spearman correlation tests. A total of 50 hub genes from specific models were further screened using the Random Survival Forests Variable Hunting (RSFVH) algorithm 40. Prognostically relevant genes were analyzed by Cox regression analysis to construct risk score models based on previous research methods. The best gene combinations or final characteristics were screened using log-rank p-values. or final features were screened by KM analysis.

### Pancan analysis

The Pancan analysis obtained gene expression data for 11,060 tumor patient samples from the TCGA Pan-Cancer (PANCAN) cohort via Xena. Samples with non-solid tumors were excluded from the analysis. The primary endpoint for survival analysis was overall survival (OS), and Cox regression-based analysis and KM analysis were performed to assess prognostic significance. To investigate the relationship between favorable prognostic metabolism-related gene sets, tumor-related genesets (EMT, cell cycle, angiogenesis), and key genes in tumor samples, z-score algorithm was employed 41.

### Cell culture and small interfering RNA (siRNA) transfection

The colon cell lines (NCM460, HT29, HCT116, SW480, RKO) and bladder cancer cell line 5637 were obtained from the American Type Culture Collection (ATCC) and cultured in Dulbecco's Modified Eagle Medium (DMEM, Gibco, USA). The DMEM was supplemented with 10% Fetal Bovine Serum (FBS, Lonsera, Australia) and a mixed antibiotic agent (100 U/mL penicillin and 100 μg/mL streptomycin). The cells were maintained at 37 °C in a humidified atmosphere containing 5% CO2. Transfection of siRNAs was performed using Lipofectamine 3000 reagent following the manufacturer's protocol. Transfection efficiency was detected by western blots. Small interfering RNA (siRNA) oligonucleotides were obtained from HanBio Technology (Shanghai, China). The siRNA sequences can be found in Table S1.

### RT-PCR and Western blots

TRI reagent was used to extract total RNA from CRC cell lines following the manufacturer's instructions (Invitrogen, USA). Total RNAs, at a concentration of 500 ng/μL, were reverse-transcribed into complementary cDNA using a two-step RT kit (Takara Biotechnology) according to the manufacturer's instructions. Finally, the amplification reaction was performed on the LightCycler 480 instrument. The target genes were amplified with GAPDH as the internal control. **Table S2** includes a list of the primer sequences used in this investigation. Total proteins were extracted using RIPA buffer (Beyotime, China) treated with phosphatase and protease suppressor. Western blotting was conducted according to the previous protocol 42. The primary antibodies used in the study were anti-GAPDH (sc-47724, Santa Cruz) and anti-GRSF1 (ab194358, Abcam).

### Cell counting kit-8 and Transwell migration assays

SW480 and RKO cells (1×10^5^/well) were cultured in 6-well plates and transfected with Si-GRSF1 or Si-NC. After 72 hours of transfection, 2000 cells were seeded into 96-well plates. The cells were cultured for 0, 24, 48, or 72 hours with Si-GRSF1 or Si-NC, followed by incubation with CCK8 solution (C0038, Beyotime, Shanghai, China) for an additional 1.5 hours. Cell viability was evaluated by measuring the optical density (OD) value at 450 nm. For cell migration assays, Transwell chambers (Corning, USA) were used. The transfected cells (4×10^4^) were suspended in 100 μl serum-free medium and placed in the top chamber, while a medium containing 10% fetal bovine serum was added to the bottom chambers. After incubating for 36 hours, the inner chambers were scrubbed, and the cells on the other side of the membrane were fixed with 4% formaldehyde solution. The cells were then stained with crystal violet and recorded under a microscope.

### Patient samples collection and Immunohistochemistry (IHC)

Fresh colon tissues were obtained from six CRC patients who underwent radical surgery and one patient who underwent a colonoscopy at the Third Affiliated Hospital of Anhui Medical University. The patients who underwent radical surgery included two cases with distant metastases and four cases in the early stage (STAGE I-II) of colon cancer without distant liver metastases. The patient who underwent colonoscopy had colon cancer with distant liver metastases. All samples were coded according to local ethical guidelines, such as those set forth in the Declaration of Helsinki, and informed consent was obtained from all patients. Colon tissues were immersed in 4% paraformaldehyde for 24 hr, embedded in araffin, sectioned, then oven-dried at 60°C for 30 min. Subsequently, GRSF1 immunohischemically detected using a primary anti-GRAF1 antibody (ab194358, Abcam) and an antirabbit secondary antibody for 30min at roomtemperature. The tissue sections were stained using a DAB Horseradish Peroxidase Color Development Kit (P0203, Beyotime, China), and then the intensity of GRSF1 staining was analyzed using ImageJ software.

## Results

### Bulk RNAseq analysis identified three metabolic pathways-related subtypes with unique genomic and transcriptional profiles in CRC

**Figure 1:**
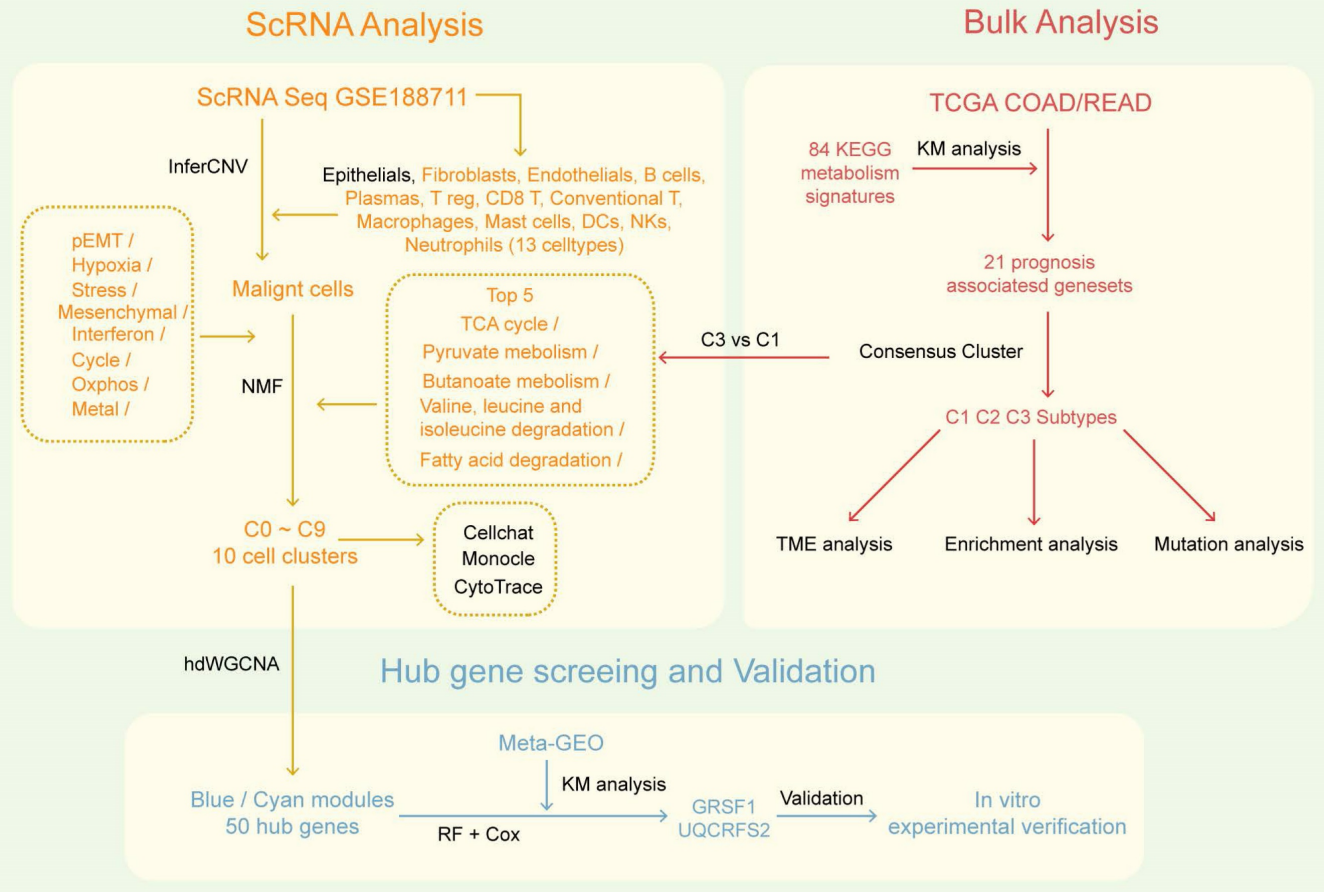
The overall flowchart of this research.

**Figure 2:**
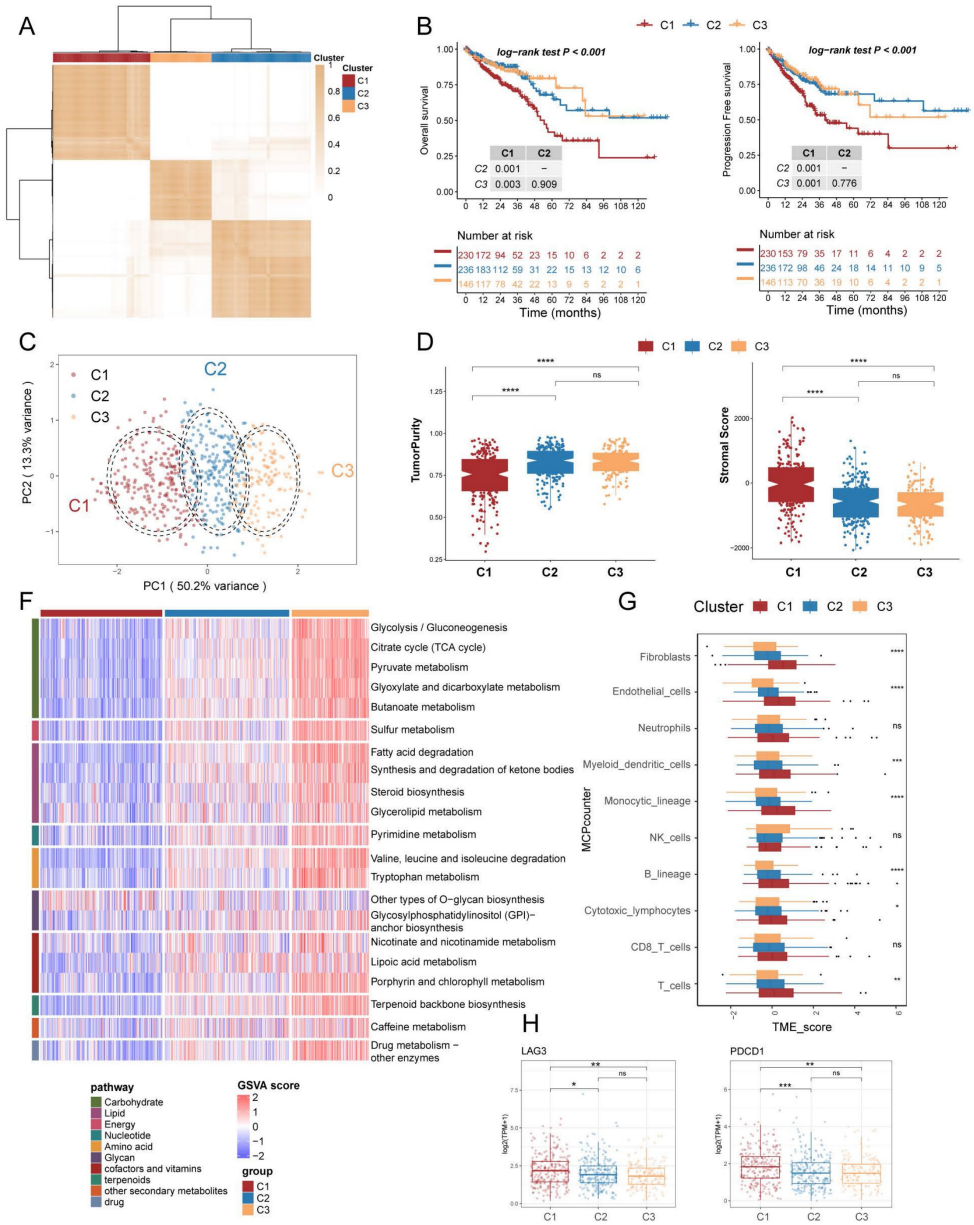
** Prognostically relevant metabolic pathway-associated subtypes identified by consensus clustering in TCGA COAD/READ cohort.** (A) The consensus matrix heat map categorizes CRC patients into 3 clusters. (B) Survival curves (overall survival, OS, and progression-free survival, PFS) demonstrate prognostic differences among the three subtypes. (C) Scatter plot illustrates the two-dimensional mapping of samples from the three subtypes based on PCA analysis. (D) Box plots display the tumor purity and stromal score of the three subtypes calculated using ESTIMATE. (E) Heatmap shows the differential expression of 21 metabolic-related pathways among the three subtypes. (F) Box plots represent the immune infiltration of ten cell types obtained from MCPcounter analysis. (G) Box plots depict the expression levels of immune checkpoint markers LAG3 and PDCD1 across the three subtypes.

The flowchart of the study is presented in **Figure 1**. To investigate the mechanism of metabolic reprogramming in CRC, we collected 85 pathways related to metabolism from the KEGG database (**Table S1**). After pathways with fewer than 2 genes were excluded, we used the remaining 84 metabolic pathway signatures in the TCGA COAD/READ cohort to score each sample by GSVA. The TCGA cohort was used to assess the prognostic value of metabolic pathways through KM survival analysis. Results showed that 21 metabolism-related pathways had prognostic significance (**Figure S2**). Subsequently, we performed consensus clustering to identify the best subtype classification (from K=2 to 4), and determined that K=3 was the optimal choice (**Figure 2A; Figure S3**). Principal component analysis (PCA) illustrates that the three clusters have unique transcriptional profiles (**Figure 2C**). Of note, patients with the C1 subtype exhibited the poorest overall survival (OS) and progression-free survival (PFS), as well as lower prognostic-associated metabolic pathway activity, except for other types of O-glycan biosynthesis (**Figure 2B, 2F**). These findings are consistent with our previous survival analysis of metabolic pathways. To determine the metabolism-related pathway typing of the tumour microenvironment (TME), we utilized the ESTIMATE and MCPcounter algorithms. Among the three subtypes, patients in subtype C1 exhibited the lowest tumour purity and the highest Stromal score (**Figure 2D**). Interestingly the MCPcounter results showed that the C1 subtype had a higher infiltration of stromal cells (fibroblasts and endothelial cells) and immune cells (Myeloid dendritic cells, Monocytic lineage, B lineage, Cytotoxic lymphocytes, and T cells) compared to the other two subtypes (**Figure 2G**). Furthermore, it was discovered that the C1 subtype exhibited increased expression of immune checkpoint molecules, specifically PDCD1 and LAG3 (**Figure 2H**). This indicates that the subtypes related to metabolic pathways may have implications for immunotherapy. Additionally, the C1 subtype is associated with advanced stage and lymph node metastatic progression relative to the other two subtypes, which correlates with the poorer prognosis observed in C1 (**Table 1**).

**Figure 3:**
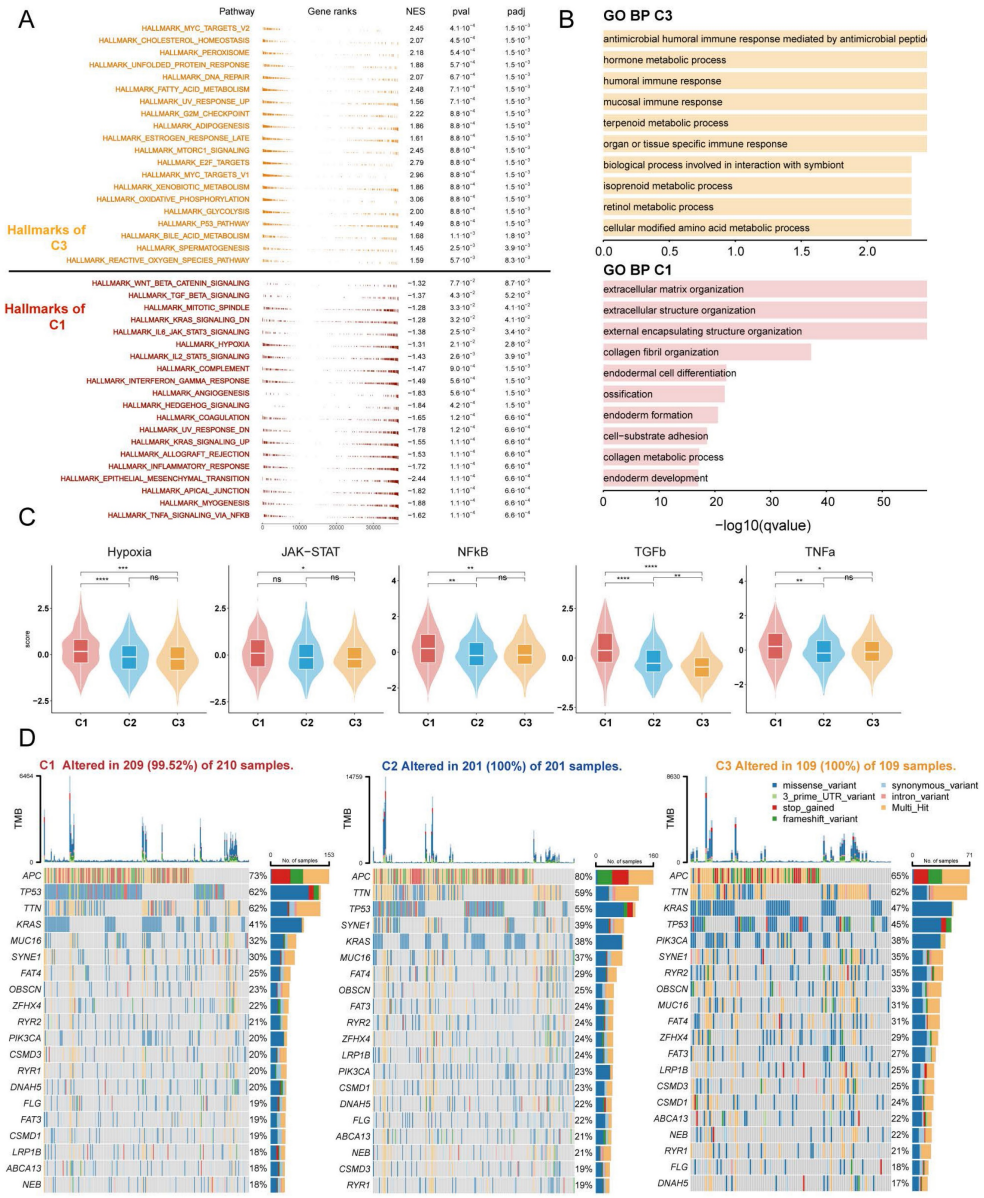
** Enrichment analysis and mutation analysis of three subtypes of colorectal cancer.** (A) The fGSEA plots of the significantly up- or down-regulated hallmark gene sets in C3-C1 (Cluster 3- Cluster 1) subtypes. (B) GO BP enrichment analyses of genes upregulated in C3 and C1. (C) Violin plots demonstrating the activity of five classical cancer-related pathways in three clusters obtained by progeny analysis. (D) The waterfall plot showing the mutation distribution of the top 20 most frequently mutated genes among three clusters.

To further investigate the distinct biological processes of each subtype, we utilised fGSEA and GO enrichment analysis to explore subtype-specific pathways. The fGSEA results indicate significant enrichment of C1 subtypes in the HALLMARK_TGF_BETA_SIGNALING, HALLMARK_EPITHELIAL_MESENCHYMAL_TRANSITION, HALLMARK_ANGIOGENESIS, and HALLMARK_COAGULATION pathways, all of which are associated with the matrix environment of the TME. In contrast, the corresponding C3 were more enriched in the HALLMARK_G2M_CHECKPOINT, HALLMARK_MITOTIC_SPINDLE, and HALLMARK_E2F_TARGETS pathways, which are associated with the cell cycle (**Figure 3A**). The GOBP enrichment analysis indicates that C1 is linked to extracellular matrix remodeling and collagen-related processes, while C3 cluster are primarily associated with immune responses and metabolic processes (**Figure 3B**). Furthermore, according to the Progeny software-based analysis, the C1 subtype exhibited a relatively higher activation of cancer-related classical pathways, such as hypoxia, JAK-STAT, NF-KB, TGFb, and TNFa (**Figure 3C**). In addition, we investigated somatic mutations in each subtype to study CRC driver genes and analyzed the top 20 genes with the highest mutation frequencies (**Figure 3D**). The mutation analysis results indicate that TP53 had a higher mutation frequency in patients with the C1 subtype, while PIK3CA had a higher mutation rate in the C3 subtype. Based on these results, we found that TME with higher stromal cell infiltration is often accompanied by lower glucose, lipid, and amino acid metabolism such as the TCA (tricarboxylic acid cycle), fatty acid degradation, and Valine, leucine and isoleucine degradation, and these high stromal and low metabolism states correlate with a poorer prognosis.

### Cellular annotation and decoding the metabolic landscape of TMEs based on scRNAseq data

**Figure 4:**
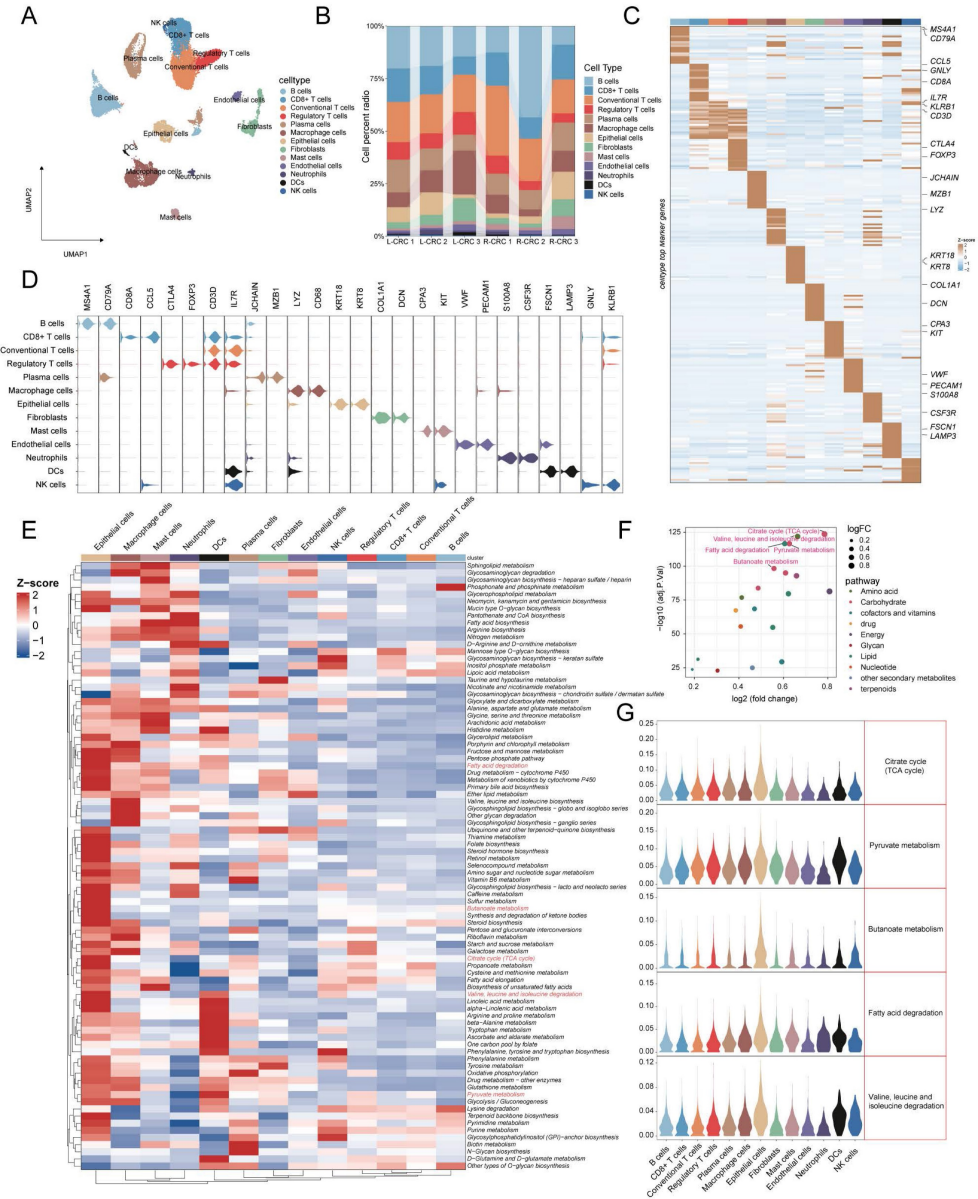
** Deciphering metabolic landscape of colorectal cancer patients at single-cell resolution** (A) UMAP visualization of 22963 cells (13 cell types) across six CRC patients. (B) Bar plot showed the 13 cell proportion among patients. (C, D) Heatmap and Violin plot showed the markers of each cell type. (E) Heatmap displaying the activity of metabolic pathways in 13 different cell types. (F) Differential analysis of metabolic pathways revealed that 20 metabolic pathways were up-regulated in C3. The top 5 pathways in the -log10 (adj.P.val) ranking are highlighted in red. (G) Violin plots indicated the metabolic pathway activity of top5 in these cell types.

To better understand metabolic heterogeneity at the cellular level in CRC, we included single-cell sequencing data from six CRC patients for analysis. After strict quality control and filtration, we obtained a total of 22,963 cells. Based on the annotation of known classical markers, 13 cell types were identified (**Figure 4A**: NK cells (markers: GNLY, KLRB1), DCs (markers: FSCN1, LAMP3), Neutrophils (markers: S100A8, CSF3R), Endothelial cells (markers: VWF, PECAM1), Mast cells (markers: KIT ,CPA3), Fibroblasts (markers: COL1A1, DCN), Epithelial cells (markers: KRT18 ,KRT8), Macrophage cells (markers: LYZ, CD68), Plasma cells (JCHAIN, MZB1), Regulatory T cells (markers: CTLA4 ,FOXP3), Conventonal T cells (markers: CD3D, IL7R), CD8 T cells (markers: CD8A ,CCL5), B cells (markers: CD79A, MS4A1)). Proportion stacked histogram showing cell proportions from 6 samples (**Figure 4B**). Violin plots and heatmaps show marker genes specific to 13 cell types (**Figure 4C, 4D**). The activity of 84 pathways in each cell type was then calculated using AUCell, and we found that epithelial cells showed abundant pathway activation compared to other cell types (**Figure 4E**). Based on differential pathway analysis between C3 and C1 subtypes, we identified 20 metabolism-related pathways that were elevated in patients with C3 subtypes, of which the TOP5 pathway (ranked by -log10adj.p.val) was the TCA cycle, Valine, leucine and isoleucine degradation, fatty acid degradation, Pyruvate metabolism and Butanoate metabolism (**Figure 4F**). The violin plot displays the top 5 pathways across all cell types, with the highest pathway activity observed in epithelial cells (**Figure 4E**). The above results indicate that epithelial cells may have a significant impact on TME through these metabolically relevant pathways.

### CRC malignant cell clusters heterogeneity and their communication with stromal cells

**Figure 5:**
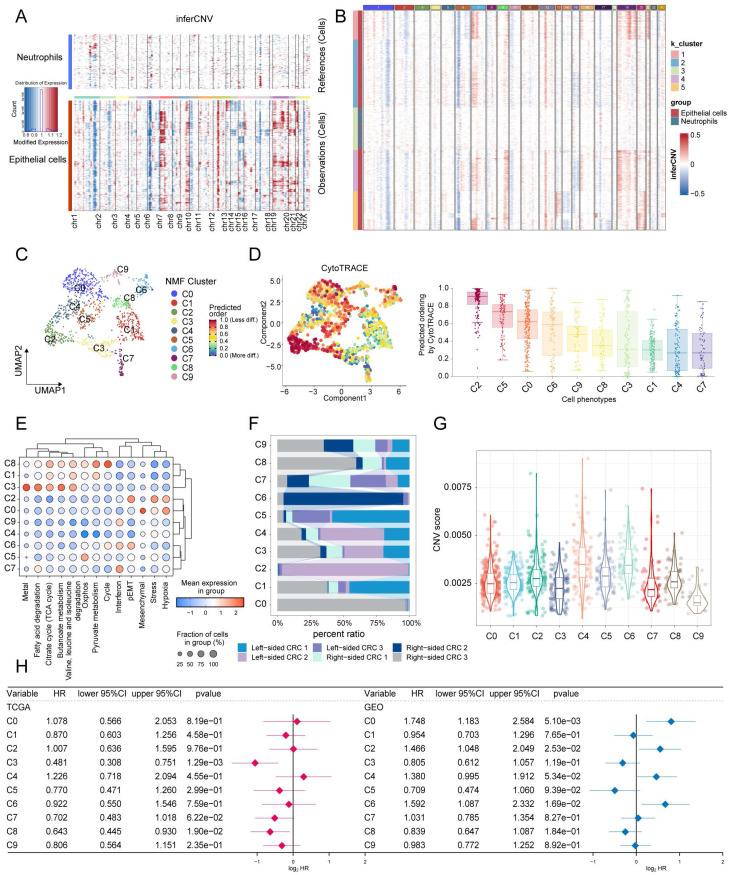
** Identification of ten tumor cell clusters by nonnegative matrix factorization (NMF). (**A) Heatmap using neutrophils as a reference to show the landscape of CNVs derived from epithelial cells. (B) K-means clustering based on CNVs reveals similarities between colon epithelial cells in Cluster 3 and neutrophils. (C) UMAP visualization of the ten tumor cell clusters. (D) Among CRC tumor cell subtypes, C2 malignancy has the highest cell stemness score per cell as determined by AUCell. (E) Dot plot displayed the scores for eight cell states and five metabolic pathways. (F) Proportion of patients in different malignant cell clusters. (G) Violin plots shows differences in CNV scores for subpopulations of malignant cells. (H) CRC Tumor cell clusters associated with patient overall survival risk in TCGA-COAD/READ (left panel) and Meta-GEO cohorts (right panel) based on Cox regression analysis.

The identification of malignant tumor cells (n = 1200) in epithelial cells was performed using InferCNV. Based on the inferred CNV matrix, we employed K-means clustering and identified five clusters. Cluster3 predominantly consisted of neutrophils and epithelial cells, which exhibited low CNVs. Based on this observation, we categorized the remaining clusters as malignant cells (**Figure 5A, B**). NMF (Non-Negative Matrix Factorization) analysis was performed using expression matrices of a total of 1096 genes from 8 tumor-associated cell states and 5 metabolism-related pathways. This analysis identified 10 distinct tumor cell clusters (**Figure 5C**). The hierarchical clustering analysis of the 13 labels revealed that clusters C1, C3, and C8 exhibited a higher metabolic pathway status. Conversely, clusters C2, C0, and C6 displayed higher levels of stress, hypoxia, epithelial-mesenchymal transition (EMT) and partial epithelial-mesenchymal transition (pEMT) (**Figure 5E**). The proportional histograms provide an illustration of the distribution of subpopulations within each cluster. It is observed that the subpopulations with high metabolic states (C1, C3, and C8) are composed of cells from multiple samples. On the other hand, the subpopulations C0, C2, and C6 have a larger proportion of cells originating from a single sample (**Figure 5F**). In addition, our findings indicate that clusters C1, C3, and C8 have relatively low cell stemness and CNV (copy number variation) scores. This suggests that these clusters may be associated with a more differentiated or less stem-like cellular phenotype, and exhibit lower levels of genomic instability in terms of copy number alterations (**Figure 5D, G**). Furthermore, we conducted GSVA on the top 100 specific genes of each tumor subclusters to estimate their relative abundance in the bulk data. In the TCGA cohort, it was observed that the C3 subgroup (HR = 0.481, 95% CI 0.308-0.751) and the C8 subgroup (HR = 0.643, 95% CI 0.445-0.930) were associated with a better prognosis. However, in the Meta-GEO cohort (GSE17536, GSE17537, GSE29621, GSE39582, and GSE72970), the C0 (HR = 1.748, 95% CI 1.183-2.584), C2 (HR = 1.466, 95% CI 1.048-2.049), and C6 (HR = 1.592, 95% CI 1.087-2.332) subgroups were associated with a worse prognosis (**Figure 5H**). Interestingly, our analysis also revealed contrasting associations between cell state signatures and prognosis in different cohorts. In the TCGA cohort, the presence of a cell cycle signature was associated with a favorable prognosis. However, in the GEO cohort, the cell state labeling related to stress, hypoxia, mesenchymal transition, and partial epithelial-mesenchymal transition (pEMT) was associated with a poorer prognosis (**Figure S4**).

**Figure 6:**
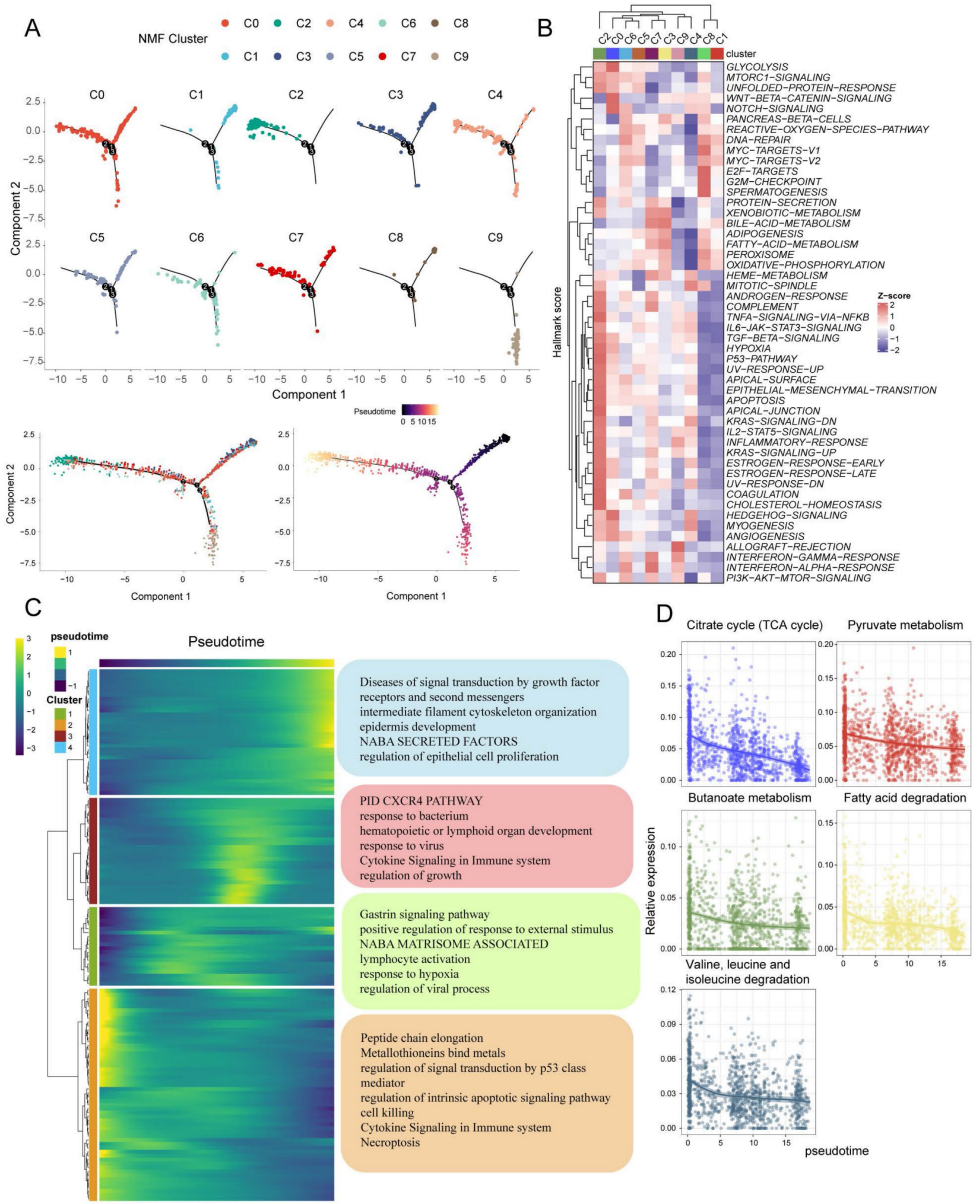
** Analysis of pseudotime trajectories for clusters of CRC tumor cells.** (A) Predicting the differentiation trajectory of subclusters of CRC tumor cells using Monocle. (B) Heatmap demonstrating Hallmark pathway scoring of CRC malignant cell subpopulations. (C) The genes that change with pseudotime have been classified into 4 main categories, and the pathways in which each category is significantly enriched by metascape. (D) Scatterplot demonstrating the decrease in 5 metabolic pathway scores with elevated pseudotime.

Subsequently, the pseudotime analysis was utilized to speculate on the developmental order of tumor cell states within the ten identified clusters. The analysis suggests that the C1 and C8 subpopulations are primarily located at the early stages of the time series, while the C2 subpopulation is predominantly found towards the later stages of the time series (**Figure 6A**). This chronological pattern suggests a potential developmental sequence of tumor cell states, with C1 and C8 potentially representing less malignant stages and C2 representing more advanced or more malignant states.

To further assess the function of each subpopulation, we used AUCell to score based on the HALLMARK geneset. The results demonstrate that the C1 and C8 subgroups exhibit similarity in their functional states. Specifically, these subgroups show relatively high activities in MYC-TARGETS, E2F-TARGETS, and G2M-CHECKPOINT pathways, while displaying low activities in TGF-BETA-SIGNALING, ANGIOGENESIS, and EMT pathways. In contrast, the C2 subpopulation showed diametrically opposed results (**Figure 6B**). Based on the chronological analysis, genes with temporal changes were categorized into four subclasses. Cluster2 genes were found to be highly expressed at the beginning of the chronological sequence and primarily associated with processes such as peptide chain elongation, metallothionein binding metals, regulation of signal transduction by p53 class mediator, regulation of intrinsic apoptotic signaling pathway, cell killing, cytokine signaling in the immune system, and necroptosis.

On the other hand, Cluster4 genes exhibited high expression mainly at the end of the chronological sequence and were significantly enriched for pathways involved in diseases of signal transduction by growth factor receptors and second messengers, intermediate filament cytoskeleton organization, epidermis development, NABA SECRETED FACTORS, and regulation of epithelial cell proliferation (**Figure 6C**). Additionally, the analysis also revealed a decrease in the scores of metabolism-related pathways with pseudotime changes (**Figure 6D**).

**Figure 7:**
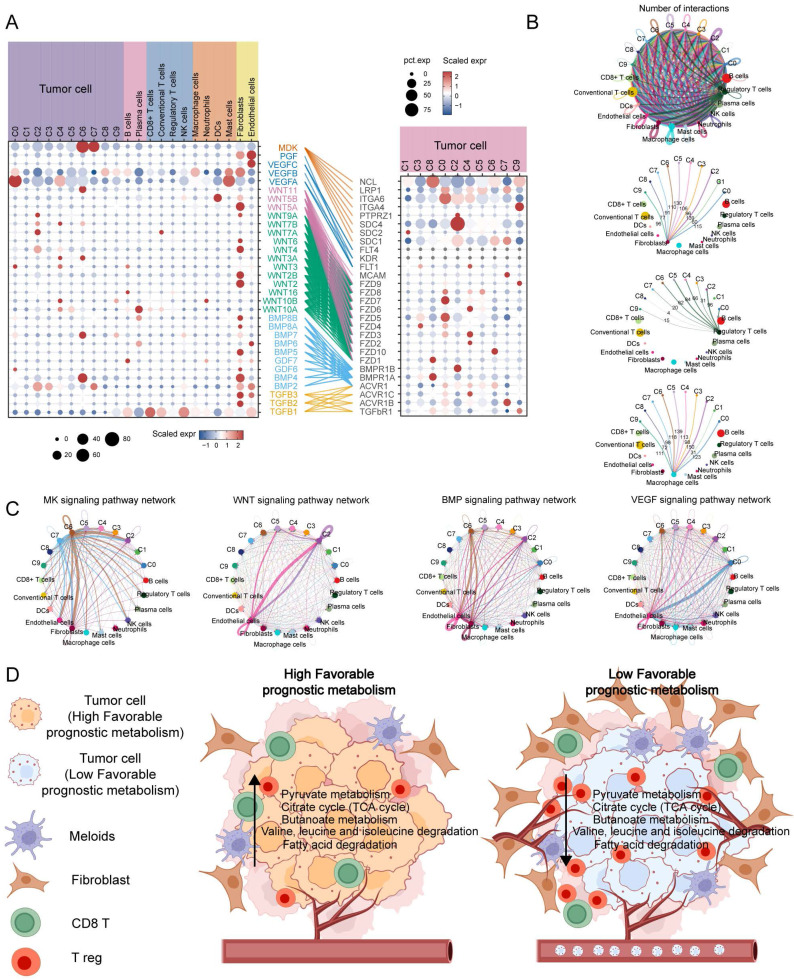
** Cell communication analysis to assess crosstalk between malignant cells and tumor microenvironment cells.** (A) Dot plots display gene expression levels of receptor-ligand pairs involved in interactions between TME and tumor clusters. (B) Overall number of interactions and crosstalk of tumor subpopulations with fibroblasts, macrophages and T reg cells, respectively. (C) Circle plots showing the interactions of MK, WNT, BMP, VEGF classical tumour-associated signalling pathways. (D) Malignant cells with a low expression of metabolic pathways associated with favorable prognosis are often accompanied by greater infiltration of Treg and fibroblasts, while promoting neovascularisation and metastasis.

To investigate the interaction between tumor cell subpopulations and TME, we utilized CellChat to infer the communication roles among different cell types. The result revealed that fibroblasts, Treg cells, and macrophages had the most frequent interactions with the C2 subpopulation, while the interactions with the C1 and C8 subpopulations were relatively infrequent (**Figure 7B**). Furthermore, it was discovered that the C6 subpopulation may have enhanced communication with stromal cells, specifically fibroblasts and endothelial cells, via the MK and BMP signaling pathways. On the other hand, the C2 and C0 subpopulations may maintain stronger crosstalk with endothelial cells through the WNT and VEGF signaling pathways, respectively (**Figure 7C**). The expression of MK, VEGF, ncWNT, WNT, BMP, and TGFB signaling pathway receptor-ligand pairs in tumor and non-tumor cells indirectly indicates possible patterns of interaction. It is noteworthy that these ligands are highly expressed on fibroblasts and endothelial cells (**Figure 7A**). In summary, **Figure 7D** shows a schematic of possible hypotheses.

### HdWGCNA and RSFVH identify hub genes in tumour clusters associated with favourable prognostic metabolic pathways

**Figure 8:**
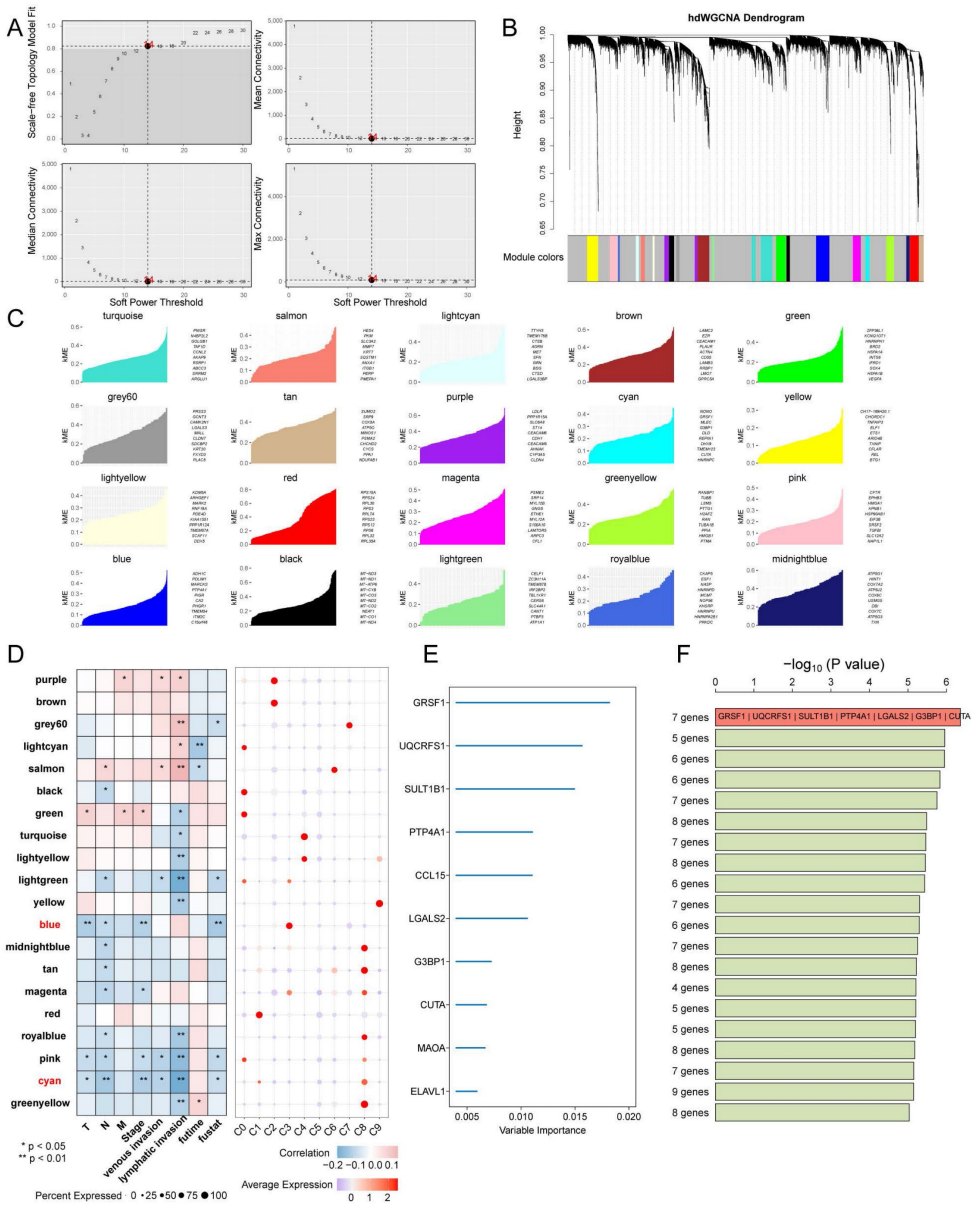
** Identification of co-expression modules and prognostically relevant hub genes in CRC tumor cells.** (A and B) Weighed gene co-expression network analysis was constructed among CRC tumor cells. (C) The top 10 eigengenes of each module are ranked by eigengene-based connectivity (kME). (D) Correlation of each module with clinical phenotypes and scoring in subpopulations of malignant cells. (E) Ten genes were screened using random survival forest analysis. (F) After conducting Kaplan-Meier analysis on 1,023 combinations, the top 20 signatures were sorted based on their p-values. The signature includes seven genes that were identified due to their relatively high -log10 p value.

**Figure 9:**
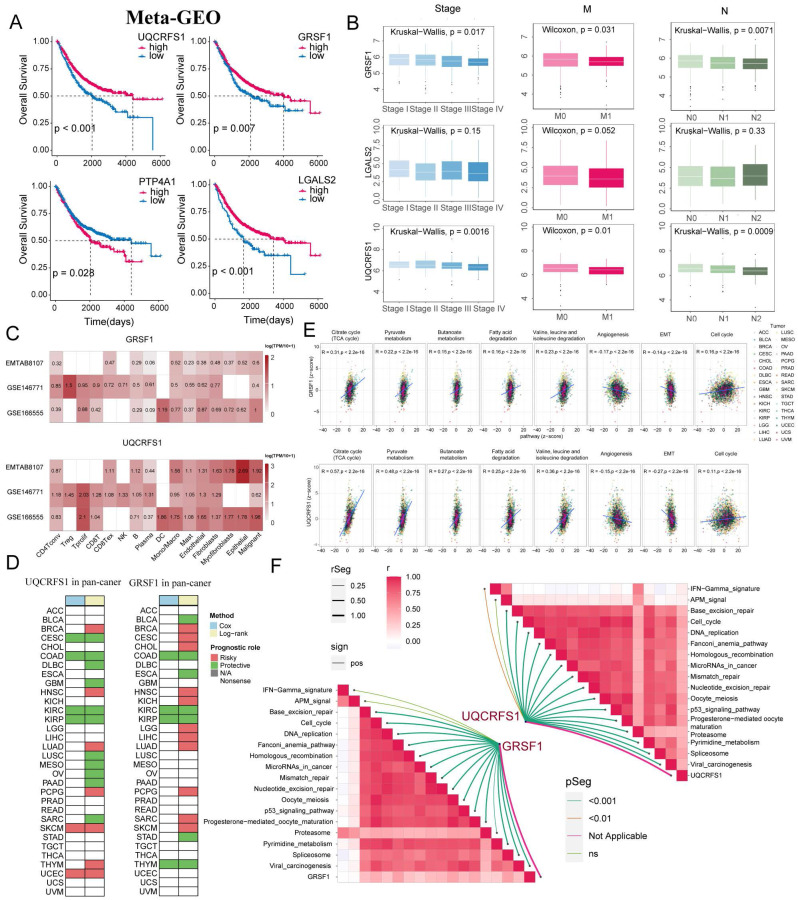
** The expression of GRSF1 and UQCRFS1 in CRC is associated with a favourable prognosis.** (A) KM analysis of UQCRFS1, GRSF1, PTP4A1, and LGALS2 in the Meta-GEO cohort. (B) Correlation of UQCRFS1, GRSF1, and LGALS2 with Stage, Lymph Node Metastasis, and Distant Metastasis. (C) Based on the TISCH2 database to identify the expression patterns of GRSF1 and UQCRFS1 in malignant and non-malignant cells across three single-cell datasets for colorectal cancer. (D) Survival analysis for UQCRFS1 and GRSF1 in pan-cancer cohort. (E) GRSF1 and UQCRFS1 correlate with five metabolic pathways and many malignant features of the tumour in the pan-cancer cohort. (F) Association of GRSF1 and UQCRFS2 with the set of immunotherapy-positive related signatures.

Next, high dimensional weighted gene co-expression network analysis (hdWGCNA) was used to identify the main molecular characteristics of each tumor cluster. With a soft threshold of 14, the scale-free network of each tumor cluster was constructed for the best connectivity and a total of 20 gene modules were identified (**Figure 8A-C**). The Blue and Cyan modules were selected based on their predominant expression in the C3 and C8 subpopulations, respectively. The Blue module showed a significant negative correlation with Stage, T, and overall survival time, while the Cyan module was negatively correlated with N, Stage, lymph node invasion, and vascular invasion (**Figure 8D**). Top 10 ranking of the 50 hub genes with the highest importance, obtained through variable screening using Random Survival Forests Variable Hunting (RSFVH) for candidate modules (**Figure 8E**). The risk models were constructed using Cox regression analysis of 1023 gene set combinations. Seven gene features (GRSF1, UQCRFS1, SULT1B1, PTP4A1, LGALS2, G3BP1, and CUTA) with the smallest p-values were identified (**Figure 8F**). In the meta-GEO cohort, KM survival analysis revealed that UQCRFS1 (p < 0.001), GRSF1 (p = 0.007), and LGALS2 (p < 0.001) were associated with a favourable prognosis (**Figure 9A**). The analysis showed that the expression of GRSF1 and UQCRFS1 decreased with the progression of tumor Stage, M-stage, and N-stage in the TCGA cohort (**Figure 9B**). **Figure 9C** shows the expression of these two candidate genes in different cell types in other single-cell sequencing datasets. Furthermore, in the PANCAN cohort consisting of 32 solid tumors, we observed that both candidate genes were positively associated with five metabolism-related pathways that have prognostic relevance, as well as the cell cycle pathway. Conversely, they showed a negative association with angiogenesis and EMT (**Figure 9D**). Additionally, these candidate genes were found to be associated with a favorable prognosis in the COAD, KIRC (kidney renal clear cell carcinoma), and KIRD (kidney renal papillary cell carcinoma) (**Figure 9E**). Correlation analysis further revealed a positive association between GRSF1 and UQCRFS1 with certain immunotherapy-positive signatures. This suggests that higher expression levels or activity of GRSF1 and UQCRFS1 may be related to increased immune response or potential sensitivity to immunotherapy in the analyzed context (**Figure 9F**).

### Down-regulation of GRSF1 inhibits CRC cell proliferation but promotes migration

To further investigate the function of GRSF1 in CRC, *in vitro* experiments were performed using CRC cells. Firstly, the mRNA expression levels of GRSF1 were examined by RT-qPCR in six cell lines.

**Figure 10:**
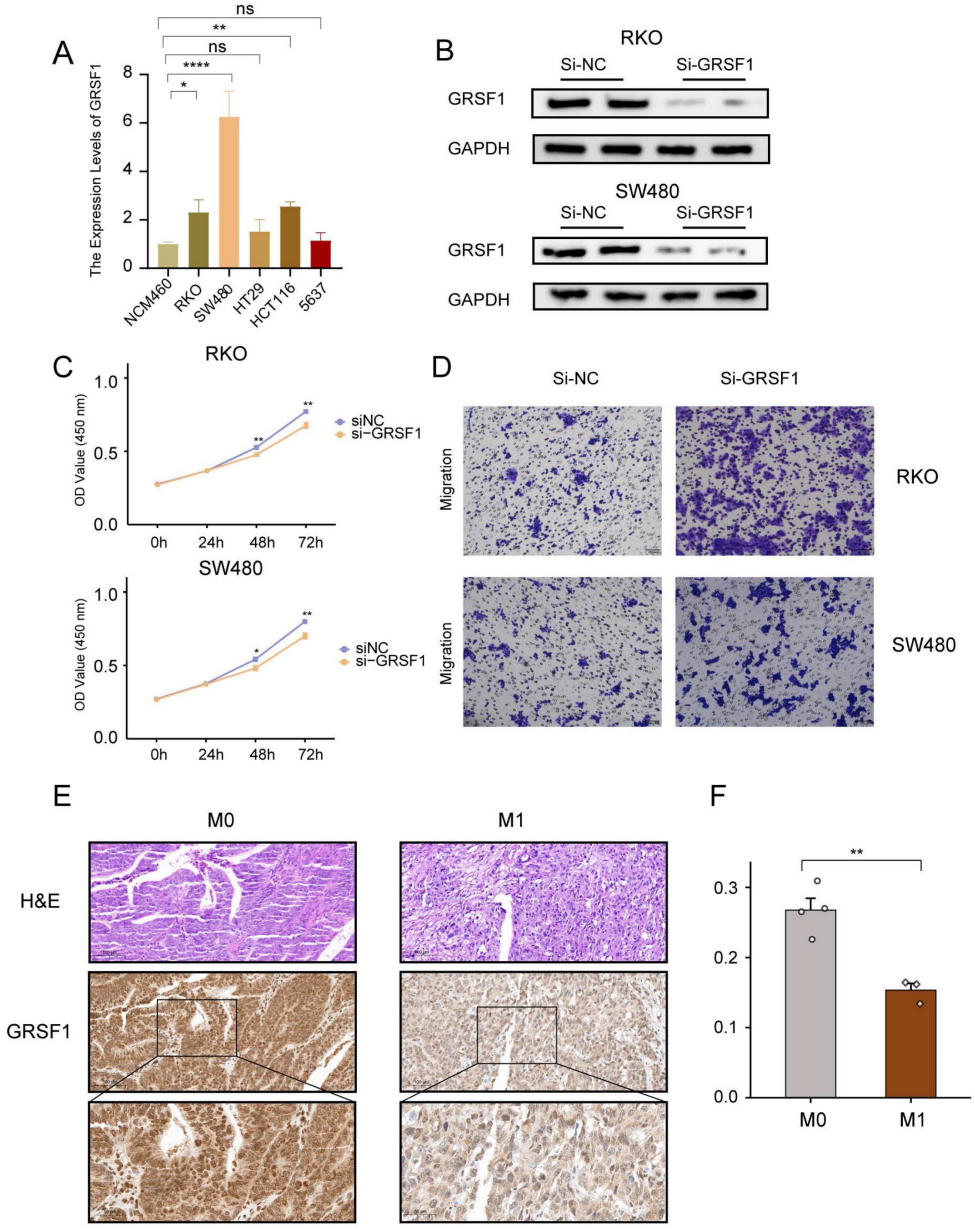
** Validation of GRSF1 through *in vitro* experiments.** (A) The mRNA expression of GRSF1 was measured in five colonrectal cell lines (NCM460, SW480, HCT116, HT29, and RKO) and blader cancer cellline 5637 using RT-qPCR. (B) Western blots reflect GRSF1 expression in RKO and SW480 cell lines treated with si-GRSF1. (C) The CCK-8 assay showed a significant reduction in cell viability after the GRSF1 knockdown. (D) The Transwell migration assay demonstrated an increased migration ability in SW480 and RKO cell lines following treatment with si-GRSF1. (E and F) Immunohistochemical images demonstrating the expression levels of GRSF1 in M0-stage and M1-stage colon cancers. (*P < 0.05, **P < 0.01).

The results showed a significant elevation of GRSF1 expression in RKO, SW480 and HCT116 compared to the normal colon cell line NCM460 (**Figure 10A**). Subsequently, the protein levels of GRSF1 expression were measured 48 hours after transfection using siRNA-mediated GRSF1 knockdown in RKO and SW480 cell lines. Western blot analysis confirmed the efficacy of GRSF1 knockdown in these cell lines (**Figure 10B**). Furthermore, the impact of GRSF1 knockdown on cell viability was assessed using the CCK8 assay. The results demonstrated a significant decrease in cell viability in RKO and SW480 cells following GRSF1 knockdown (P < 0.01) (**Figure 10C**). This suggests that GRSF1 may play an essential role in promoting the proliferation of CRC cell lines. Additionally, Transwell migration experiments were conducted to evaluate the effect of GRSF1 knockdown on cell migration. The results revealed that GRSF1 knockdown significantly promoted the migration of RKO and SW480 cells (**Figure 10D**). Immunohistochemistry analysis reveals diminished expression of GSRF1 in colon cancer specimens classified as stage M1 when contrasted with those at stage M0 (**Figure 10E, F**). These findings collectively suggest that GRSF1 may have functional significance in CRC, including its involvement in cell proliferation and migration processes.

## Discussion

In recent years, the advancement of multi-omics technologies has significantly improved the diagnosis and treatment of CRC. Despite these advancements, it is still concerning that approximately 60% of CRC patients eventually develop metastases 43. Moreover, the use of multiple forms of combination therapy often leads to the development of resistance, further complicating the management of CRC metastases 44, 45. As a result, CRC metastases remain the primary cause of cancer-related deaths. Indeed, metabolic reprogramming plays a crucial role in tumor progression and metastasis and is considered a fundamental hallmark of cancer 46. Cancer cells often undergo metabolic rewiring to meet their high energy and nutrient demands required for rapid cell division 47. Several proteomic and metabolomic studies have revealed that many metabolic pathways are altered in colorectal tumors compared to normal mucosa 48, 49. However, targeting or combining metabolic pathways in tumor cells presents a significant challenge due to the complexity of tumor metabolism. The development of single-cell technology provides a new vision for our understanding of tumor and TME metabolism.

Our study provides comprehensive insights into the metabolic heterogeneity within the TME of CRC, significantly advancing our understanding of metabolic reprogramming in cancer progression beyond what has been achieved in some previous studies 50-52. Unlike previous studies that have broadly characterized metabolic pathways associated prognosis model in CRC, our research utilizes advanced multi-omics approaches, including bulk RNAseq and scRNAseq analyses, to dissect these pathways at a more granular, cellular level. This allows us to identify distinct metabolic subtypes and characterize cellular annotations, which are crucial for tailoring personalized treatment strategies. Initially, we collected 85 metabolism-related pathways from the KEGG database and scored them by GSVA for the TCGA CRC cohort. The results showed prognostic significance for 21 metabolic related pathways. Through clustering analysis, we identified three optimal subtype classifications, where the C1 subtype exhibited the poorest OS and PFS. Moreover, the C1 subtype displayed higher activation of classic pathways such as Hypoxia, JAK-STAT, NF-KB, TGFb, and TNFa, which promote tumorigenesis and development 53-57. Furthermore, our investigation into the TME revealed that the C1 subtype displayed higher infiltration of stromal cells (fibroblasts and endothelial cells). Further exploration using enrichment analysis highlighted the significance of extracellular matrix remodeling and collagen-related processes in the C1 subtypes. In comparison, the C3 subtype was more enriched in G2M checkpoint, mitotic spindle, and E2F targets pathways associated with the cell cycle 58. Additionally, increased expression of immune checkpoint molecules, specifically PDCD1 and LAG3, was observed in the C1 subtype, suggesting potential implications for immunotherapy.

To gain a deeper understanding of metabolic heterogeneity at the cellular level in CRC, we incorporated scRNA seq data from six CRC patients. Differential pathway analysis between the C3 and C1 subtypes revealed 20 metabolism-related pathways that were elevated in patients with the C3 subtype. These pathways included the TCA cycle, Valine, leucine and isoleucine degradation, fatty acid degradation, Pyruvate metabolism, and Butanoate metabolism. Interestingly, the top five pathways demonstrated the highest activity in epithelial cells. The famous Warburg effect refers to the tendency of cancer cells to utilize anaerobic glycolysis rather than oxidative phosphorylation even in the presence of an adequate supply of oxygen 59. And both the TCA cycle as well as Pyruvate metabolism were significantly associated with this effect. It has been shown that NCAPD3 enhances the Warburg effect in colon cancer, including the enhancement of cellular aerobic glycolysis and the inhibition of TCA cyclic flux, which promotes tumor development 60. In addition, butyrate is mainly produced by the gut microbiota during colonic fermentation. Fewer butyrate-producing bacteria were detected in the microbiota of CRC patients than in controls 61. Numerous studies have shown that butyrate exerts anticancer activity in colorectal cancer by affecting multiple signaling pathways 62-64. We performed InferCNV analysis and K-means clustering, which resulted in the identification of five clusters. Among them, Cluster 3 consisted predominantly of neutrophils and epithelial cells. The remaining clusters were categorized as malignant cells. NMF analysis was then conducted, revealing ten distinct tumor cell clusters. Hierarchical clustering analysis showed that clusters C1, C3, and C8 exhibited higher metabolic pathway statuses, while clusters C0, C2, and C6 displayed higher levels of stress, hypoxia, EMT, and pEMT. We further investigated interactions between tumor cell subgroups and the TME, finding that fibroblasts, Treg cells, and macrophages most frequently interacted with the C2 subgroup, but relatively less with C1 and C8 subgroups. Interactions between tumor cells and adjacent CAFs (Cancer-associated fibroblasts) can influence tumor progression and treatment resistance, where possible mechanisms include induction of epithelial-to-mesenchymal transition (EMT), stemness-associated programs, and metabolic reprogramming of tumor cells 65. Recent studies have also shown that CAFs secreted exosomal miR-92a-3p promote metastasis and chemotherapy resistance of CRC 66. Moreover, we found that the C6 subgroup might enhance communication with stromal cells (especially fibroblasts and endothelial cells) through the MK and BMP signaling pathways. Whereas C2 and C0 subpopulations have strong communication with endothelial cells through WNT and VEGF pathways, respectively. BMP and WNT signaling pathways crosstalk in CRC, Loss of SMAD4 alters BMP signaling activates wnt signalingto promote CRC cell metastasis 67, 68. The vascular endothelial growth factor (VEGF) family and its receptors are considered to be the most prominent regulators of angiogenesis, affecting tumor progression and metastasis 69. Prognostic analysis also indicated that the C0, C2, and C6 subgroups were associated with a worse prognosis in the Meta-GEO cohort, whereas the C3 and C8 subgroups were associated with a better prognosis in the TCGA cohort. It is worth mentioning that C1 and C8 exhibited relatively low cell stemness and CNV scores. In summary, we suggest that tumor cell subpopulations are accompanied by changes in top5 metabolism-related prognostic pathway activity with different TME and pathway crosstalk.

Moreover, by employing the hdWGCNA technique, we were able to identify 20 gene modules that correlate with clinical features of CRC. Particularly, the blue and cyan modules predominantly expressed in the C3 and C8 subgroups respectively, offer noval biomarkers for predicting the metastatic potential of CRC subtypes. Through RSFVH and external cohorts validation, we identified the key gene GRSF1. This discovery emphasizes the central role of GRSF1 in modulating the cancer metabolism landscape, positioning it as a key player worth further investigation. Furthermore, in light of our findings, in the PANCAN cohort, GRSF1 not only associates positively with metabolism-related and cell cycle pathways but also shows negative association with angiogenesis and EMT, positioning it as a key player in modulating the cancer metabolism landscape. It was also associated with a favorable prognosis in KIRC and KIRD. Correlation analysis showed a positive correlation between GRSF1, and certain immunotherapy-positive features, suggesting that it may facilitate immunotherapy response. Various studies have shown the oncogenic effects of GRSF1 in gastric cancer, cervical cancer, lung adenocarcinoma, triple-negative breast cancer, and hepatocellular carcinoma 70-74.

Interestingly in our study, GRSF1 plays a dual role in CRC including promotion of proliferation and inhibition of metastasis. A number of cancer-related signaling molecules have been shown to play a dual role in the development and progression of cancer. For example, Epcam is prognostically favorable in both KIRC and endometrial cancer, but has been shown to promote proliferation and inhibit invasion and migration *in vitro* 75, 76. Furthermore, FBXO22 has a paradoxical role in breast cancer, promoting breast tumor cell proliferation while preventing EMT and metastasis 77. Similarly, SnoN, an important negative regulator of TGFβ signaling, plays a role in suppressing EMT and promoting proliferation in mammalian 78. Based on these findings, we speculate that GRSF1 may play a role in promoting tumor clone formation early in tumorigenesis. And when the tumor metastasizes or progresses, the expression of GRSF1 would be reduced through some as yet undefined mechanisms. Our study suggests that targeting GRSF1 in CRC may not be a good therapeutic approach. However, our study has several limitations. Firstly, Due to the lack of sufficient clinical information on the samples in the public databases TCGA and GEO, it is possible that other disease states in some patients may also affect the metabolic profile of tumor cells. The mechanism by which GRSF1 promotes tumor progression needs to be further explored and corresponding *in vivo* experiments are lacking as this study only found inhibition of migration in CRC cell lines *in vitro* Moreover, our findings of GRSF1 expression and its correlation with clinical features require validation in a larger patient cohort. The process of GRSF1 crosstalk with immune cells in TME also requires further experimental validation.

Overall, the detailed elucidation of metabolic heterogeneity and its implications in CRC provided by our study not only fills gaps left by previous research but also sets the stage for novel therapeutic interventions that are finely tuned to the metabolic nuances of individual tumors. Through bulk RNAseq and scRNAseq analyses, we identified distinct metabolic subtypes, characterized cellular annotations, elucidated communication patterns between malignant cells and stromal cells, and identified hub gene GRSF1 associated with favorable prognostic metabolic pathways. This underlines the importance of our findings for understanding the intricate nature of the metabolic landscape in CRC and for guiding future efforts to develop targeted therapies. Our findings may contribute to a better understanding of the metabolic landscape in CRC and may have implications for the development of targeted therapies and immunotherapies.

## Supplementary Material

Supplementary figures and tables.

## Figures and Tables

**Table 1:** Clinical features of the three metabolic subtypes of TCGA CRC

Characteristics	C1(N=231)	C2(N=236)	C3(N=146)	pvalue
**Sex**				0.45
FEMALE	106	117	63	
MALE	125	119	83	
**T**				0.1
T1-2	36	53	34	
T3-4	194	182	112	
**N**				4.50E-04
N0	110	139	99	
N1-2	120	95	47	
**M**				0.29
M0	158	179	118	
M1	37	30	18	
**Stage**				1.50E-03
Stage I-II	106	130	94	
Stage III-IV	117	99	47	
**lymphatic invasion**				0.32
NO	118	134	76	
YES	95	80	52	
**Venous invasion**				0.05
NO	143	158	101	
YES	61	49	22	
**Age**				0.4
≥65	137	138	95	
<65	94	98	51	
**BMI**				0.23
Normal weight	37	38	18	
obese	43	34	12	
overweight	56	43	18	
underweight	5	0	0	

## Abbreviations

CRC: Colorectal Cancer
GEO: Gene Expression Omnibus
TCGA: The Cancer Genome Atlas
GSVA: Gene Set Variation Analysis
CNV: Copy number variation
NMF: Non-negative Matrix Factorization
RSFVH: Random Survival Forests Variable Hunting
hdWGCNA: high dimensional weighted gene co-expression network analysis
UQCRFS1: Ubiquinol-Cytochrome C Reductase, Rieske Iron-Sulfur Polypeptide 1
GRSF1: G-Rich RNA Sequence Binding Factor 1
TPM: transcripts per million
scRNA-seq: single-cell RNA sequencing
PCA: principal component analysis
UMAP: Unified Mobility Approximation and Projection
TME: tumor microenvironment
FGSEA: Fast Genome Enrichment Analysis
GOBP: gene ontology biological process
KEGG: Kyoto Encyclopedia of Genes and Genomes
EMT: epithelial-mesenchymal transition
KIRC: kidney renal clear cell carcinoma
KIRD: kidney renal papillary cell carcinoma
CAFs: Cancer-associated fibroblasts
