# Supplementary material for: Metabolic Heterogeneity of Tumor Cells and its Impact on Colon Cancer Metastasis: Insights from Single-Cell and Bulk Transcriptome Analyses
Source: J Cancer. 2024 Jun 3;15(13):4175–96. doi: 10.7150/jca.94630 (PMC11212087; doi:10.7150/jca.94630)
Supplement: Supplementary file 1 — Supplementary figures and tables. [file jcav15p4175s1.pdf]

Supplementary Figure 1. Integration of GEO datasets batch correction.

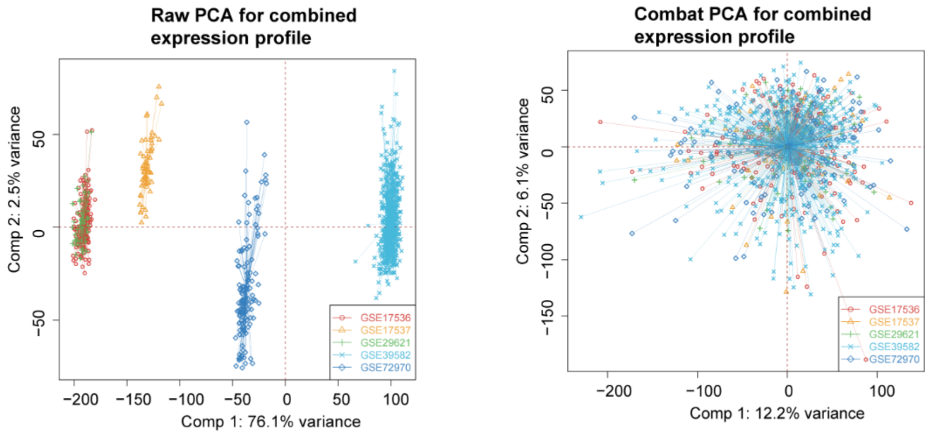

Supplementary Figure 2. Survival analysis of 21 metabolism-related pathways in the TCGA CRC cohort.

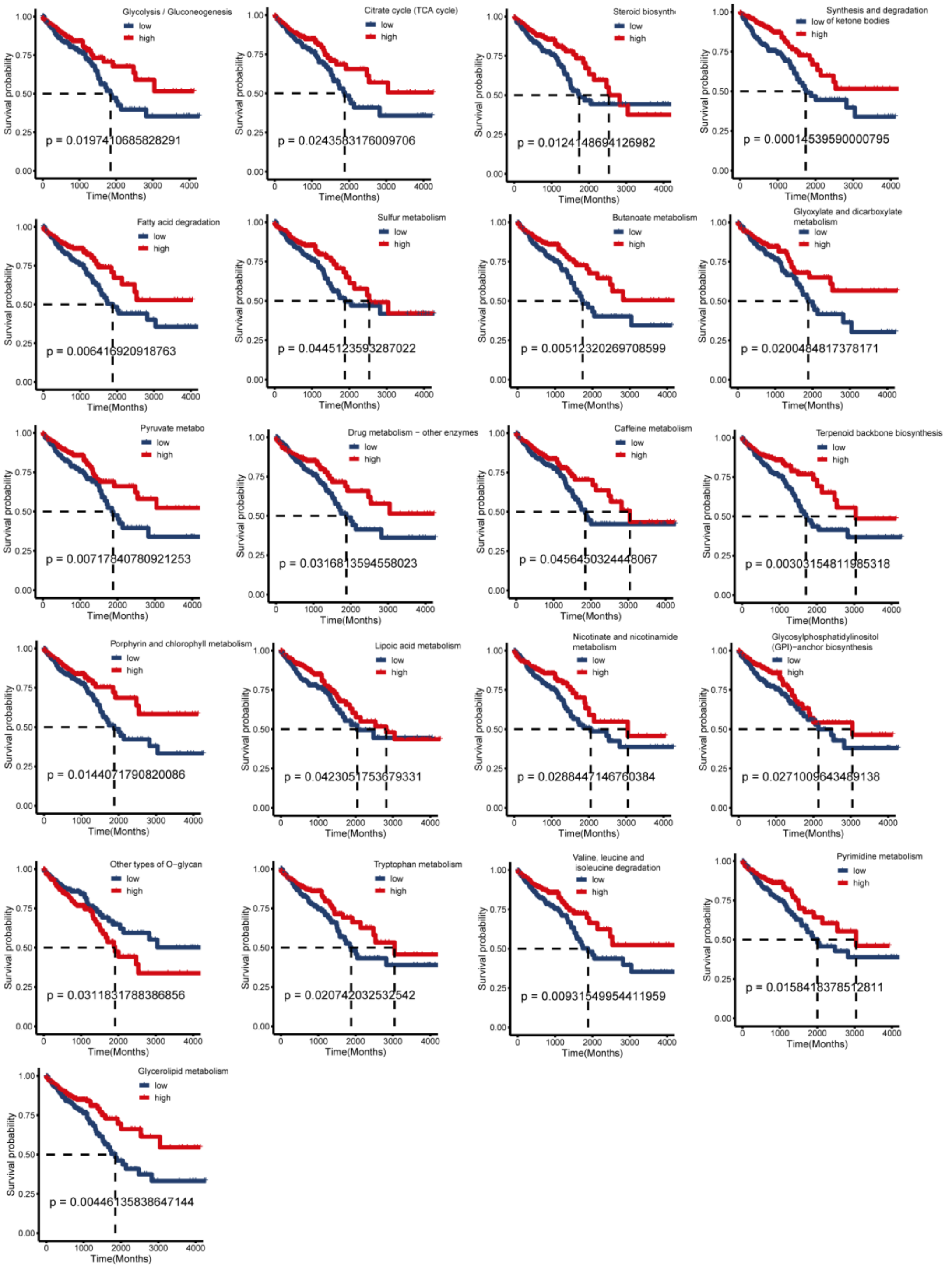

Supplementary Figure 3. Analysis of consensus clustering was performed for K=2 to K=4.

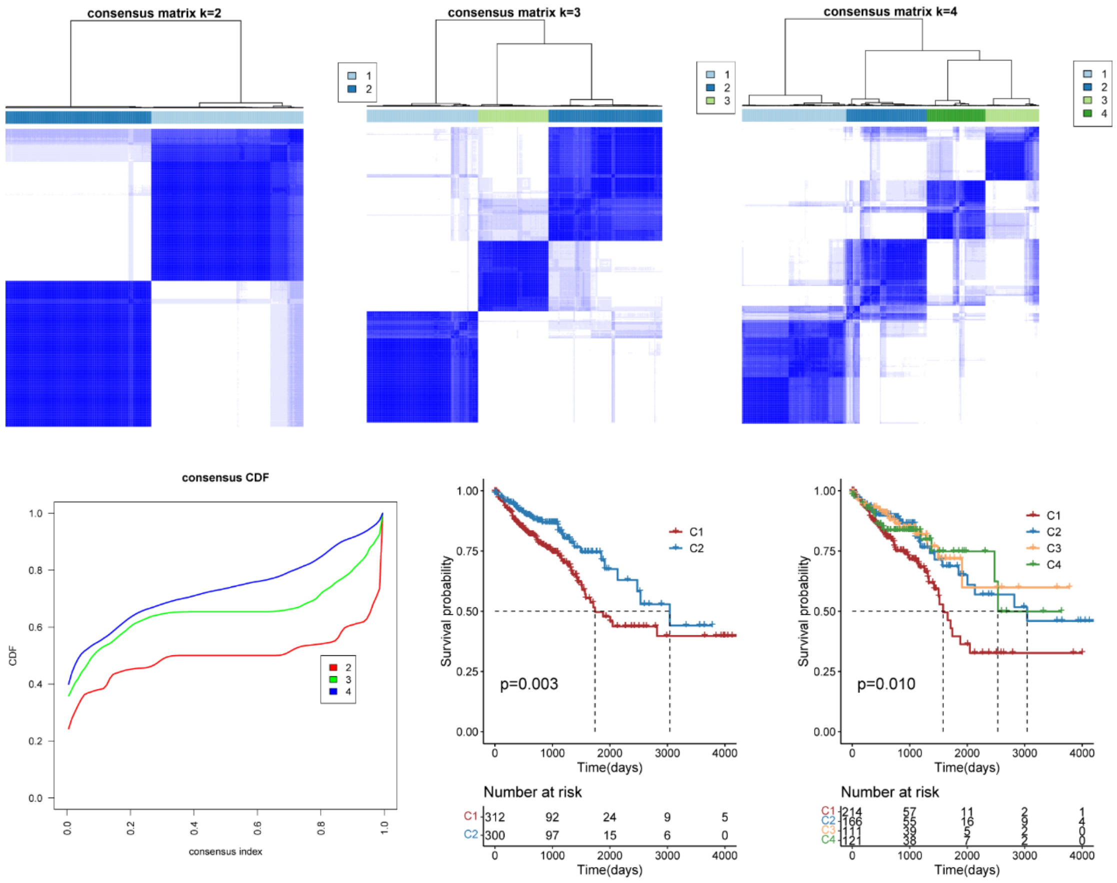

Supplementary Figure 4. Cox regression analysis of 8 cell states in TCGA and Meta-GEO cohorts.

TCGA (COAD/READ)

Variable	HR	lower 95%CI	upper 95%CI	pvalue
Cycle	0.603	0.400	0.909	1.57e−02
Stress	0.835	0.427	1.633	5.99e−01
Interferon	0.823	0.549	1.234	3.46e−01
Hypoxia	1.698	0.985	2.928	5.67e−02
Oxphos	0.964	0.682	1.361	8.33e−01
Metal	1.089	0.732	1.621	6.73e−01
Mesenchymal	1.192	0.857	1.659	2.97e−01
pEMT	0.969	0.550	1.707	9.13e−01

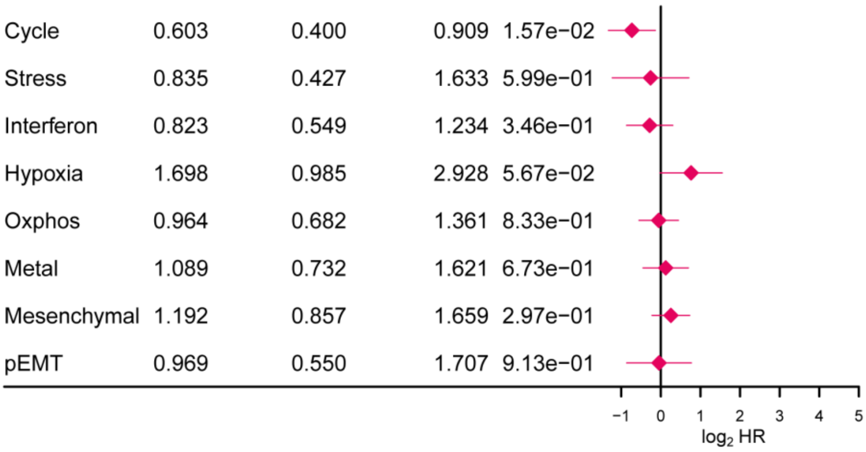

GEO (GSE17536 GSE17537 GSE29621 GSE39582 GSE72970)

Variable	HR	lower 95%CI	upper 95%CI	pvalue
Cycle	0.884	0.697	1.121	3.09e−01
Stress	1.840	1.282	2.642	9.46e−04
Interferon	0.948	0.744	1.207	6.65e−01
Hypoxia	1.658	1.209	2.273	1.71e−03
Oxphos	0.840	0.660	1.069	1.56e−01
Metal	1.185	0.953	1.472	1.26e−01
Mesenchymal	1.355	1.120	1.640	1.80e−03
pEMT	1.718	1.220	2.420	1.95e−03

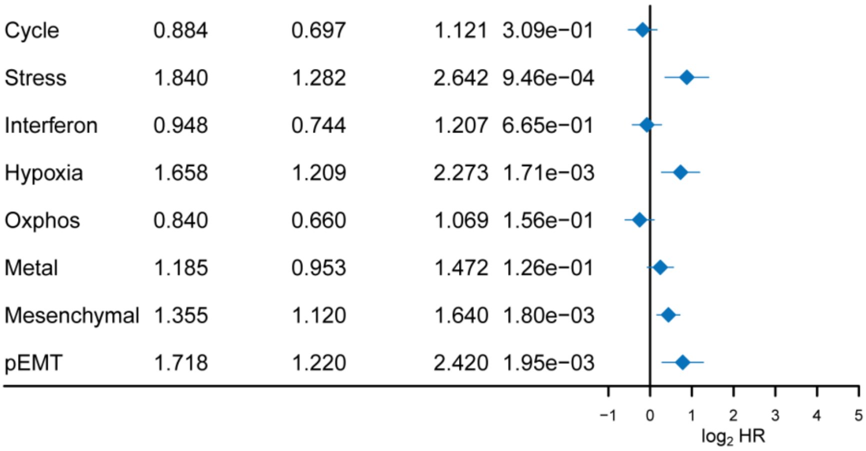

Supplementary Table 1 Gene list of the 85 human metabolic pathways in the KEGG database

Genepathways	hsa code
Glycolysis / Gluconeogenesis	10
Citrate cycle (TCA cycle)	20
Pentose phosphate pathway	30
Pentose and glucuronate interconversions	40
Fructose and mannose metabolism	51
Galactose metabolism	52
Ascorbate and aldarate metabolism	53
Starch and sucrose metabolism	500
Amino sugar and nucleotide sugar metabolism	520
Pyruvate metabolism	620
Glyoxylate and dicarboxylate metabolism	630
Propanoate metabolism	640
Butanoate metabolism	650
Inositol phosphate metabolism	562
Oxidative phosphorylation	190
Nitrogen metabolism	910
Sulfur metabolism	920
Fatty acid biosynthesis	61
Fatty acid elongation	62
Fatty acid degradation	71
Synthesis and degradation of ketone bodies	72
Steroid biosynthesis	100
Primary bile acid biosynthesis	120
Steroid hormone biosynthesis	140
Glycerolipid metabolism	561
Glycerophospholipid metabolism	564
Ether lipid metabolism	565
Sphingolipid metabolism	600
Arachidonic acid metabolism	590
Linoleic acid metabolism	591
alpha-Linolenic acid metabolism	592
Biosynthesis of unsaturated fatty acids	1040
Purine metabolism	230
Pyrimidine metabolism	240
Alanine, aspartate and glutamate metabolism	250
Glycine, serine and threonine metabolism	260
Cysteine and methionine metabolism	270
Valine, leucine and isoleucine degradation	280
Valine, leucine and isoleucine biosynthesis	290

Lysine degradation	310
Arginine biosynthesis	220
Arginine and proline metabolism	330
Histidine metabolism	340
Tyrosine metabolism	350
Phenylalanine metabolism	360
Tryptophan metabolism	380
Phenylalanine, tyrosine and tryptophan biosynthesis	400
beta-Alanine metabolism	410
Taurine and hypotaurine metabolism	430
Phosphonate and phosphinate metabolism	440
Selenocompound metabolism	450
D-Glutamine and D-glutamate metabolism	471
D-Arginine and D-ornithine metabolism	472
Glutathione metabolism	480
N-Glycan biosynthesis	510
Mucin type O-glycan biosynthesis	512
Mannose type O-glycan biosynthesis	515
Other types of O-glycan biosynthesis	514
Glycosaminoglycan biosynthesis - chondroitin sulfate / dermatan sulfate	532
Glycosaminoglycan biosynthesis - heparan sulfate / heparin	534
Glycosaminoglycan biosynthesis - keratan sulfate	533
Glycosaminoglycan degradation	531
Glycosylphosphatidylinositol (GPI)-anchor biosynthesis	563
Glycosphingolipid biosynthesis - lacto and neolacto series	601
Glycosphingolipid biosynthesis - globo and isoglobo series	603
Glycosphingolipid biosynthesis - ganglio series	604
Other glycan degradation	511
Thiamine metabolism	730
Riboflavin metabolism	740
Vitamin B6 metabolism	750
Nicotinate and nicotinamide metabolism	760
Pantothenate and CoA biosynthesis	770
Biotin metabolism	780
Lipoic acid metabolism	785
Folate biosynthesis	790
One carbon pool by folate	670
Retinol metabolism	830
Porphyrin and chlorophyll metabolism	860
Ubiquinone and other terpenoid-quinone biosynthesis	130
Terpenoid backbone biosynthesis	900
Caffeine metabolism	232
Neomycin, kanamycin and gentamicin biosynthesis	524

Metabolism of xenobiotics by cytochrome P450	980
Drug metabolism - cytochrome P450	982
Drug metabolism - other enzymes	983

Supplementary Table 2 Nucleic acid sequences of SiRNAs and primers

**Oligonucleotides**	**Nucleotides sequence (5' -3')**
**SiRNA**	
siRNA NC	UUCUCCGAACGUGUCACGU TT (sense)<br>ACGUGACACGUUCGGAGAA TT(anti-sense)
GRSF1（h）-si	GGAUGUGCAGAAAGCCUUAdTdT(sense)<br>UAAGGCUUUCUGCACAUCCdTdT(anti-sense)
**Primer**	
GRSF1	TGAGTATGAATTGGCCCCGT (forward)<br>ATCACCCCTTCGTTTCCCAT (reverse)
GAPDH	GTCTCCTCTGACTTCAACAGCG(forward)<br>ACCACCCTGTTGCTGTAGCCAA(reverse)
